# Supplementary material for: WordCommentsAnalyzer: A windows software tool for qualitative research
Source: F1000Res. 2018 Sep 4;7:536. Originally published 2018 May 3. [Version 2] doi: 10.12688/f1000research.14819.2 (PMC6137411; doi:10.12688/f1000research.14819.2)
Supplement: Supplementary file 3 [file f1000research-7-17614-s0002.tgz › b2a79496-4ada-47c4-9682-a99ce867836d.zip!/Eight Iranian Health Journals/Yafteh _16.docx]

© Yafteh  . This work is licensed under CC BY 4.0 (https://creativecommons.org/licenses/by/4.0/).

Volume & Issue: دوره 16 - شماره 2

Title: پراکندگی و تعیین فون کنه های سخت و نرم شهرستان خرم آباد، استان لرستان در سال‌ 91-1390

http://yafte.lums.ac.ir/article-1-1663-fa.html

مقدمه: کنه ها انواع عوامل بیماریزا از قبیل آربوویروس ها، باکتری ها و انگل ها را به انسان و حیوانات انتقال می دهند. اهمیت آنها به عنوان ناقل ویروس تب خونریزی دهنده کریمه کنگو به انسان، به اثبات رسیده است. لذا شناسایی فون کنه ها در یک منطقه، در پایش برنامه های کنترل آن ها و در نتیجه پیشگیری از بیماریها بسیار حائز اهمیت می باشد. مواد و روش‌ها: دام ها و کنه های روستاهای شهرستان خرم‌آباد جمعیت مورد مطالعه ما را شامل می شدند. از 40 روستا کنه‌ها از دام‌های اهلی جدا شدند و با استفاده از کلیدهای تشخیصی معتبر مانند کلید تشخیصی کنه‌های سخت روسیه و سایر کلیدهای موجود اقدام به تشخیص نمونه ها در سطح جنس و گونه گردید. یافته‌ها: از 800 دام مورد بررسی تعداد 3156 عدد کنه سخت جدا گردید از کل کنه های صید شده تعداد 2319 کنه (73%) در گوسفندان، 823 عدد (26%) در بزها و 14 کنه (1%) در گاوها صید شدند. از نظر تنوع کنه های صید شده تعداد چهار جنس هیالوما، درماسنتور، همافیزالیس و ریپی سفالوس از دام ها جدا گردید. از جنس هیالوما سه گونه (هیالوما آناتولیکوم، هیالوما آسیاتیکوم و هیالوما مارژیناتوم)، از جنس درماسنتور گونه مارژیناتوس، از جنس همافیزالیس گونه سولکاتا و از جنس ریپی سفالوس گونه سنگووینوس شناسایی گردیدند. بحث و نتیجه‌گیری: در این بررسی چهار جنس و شش گونه از کنه های ایگزودیده از دامها جدا گردید. با توجه به اهمیت این گونه ها در انتقال بیماری ها به انسان، مسئولین بهداشتی و سازمان های ذیربط باید اقدامات بهداشتی مناسب جهت کنترل و مبارزه با این انگل های خارجی را به عمل آورند.

Title: فراوانی بتالاکتامازهای وسیع الطیف در بین ایزوله های اشریشیاکلی یوروپاتوژنیک بیمارستان ابن سینای شهرستان دلفان، استان لرستان

http://yafte.lums.ac.ir/article-1-1664-fa.html

مقدمه: شایع ترین علت مقاومت به آنتی بیوتیک های بتالاکتام در اشریشیاکلی، تولید آنزیم های بتالاکتاماز می باشد. متأسفانه دسته ای از این آنزیم ها، به نام "بتالاکتاماز های وسیع الطیف" (ESBLs) قادرند سفالوسپورین های نسل سوم جدید و آنتی بیوتیک آزترونام را نیز غیر فعال کنند. مطالعات متعددی در ایران و سایر نقاط جهان، فراوانی ESBLs را گزارش کرده اند. لذا هدف از این مطالعه، تعیین حساسیت میکروبی و فراوانی ESBLs در بین ایزوله های اشریشیاکلی یوروپاتوژنیک شهرستان دلفان، لرستان در سال1391 بود. مواد و روش‌ها: در مطالعه حاضر، 100 ایزوله اشریشیاکلی یوروپاتوژنیک از بیماران بستری در بیمارستان ابن سینای شهرستان دلفان جمع آوری و تعیین هویت شد. حساسیت آنتی بیوتیکی ایزوله ها به روش انتشار از دیسک بر روی محیط مولر - هینتون آگار ارزیابی گردید. بررسی فنوتیپی تولید ESBLs در بین ایزوله های مورد آزمایش، با استفاده از از دیسک های سفوتاکسیم، سفتازیدیم و سفپودوکسیم به تنهایی و در ترکیب با کلاولونیک اسید (دیسک ترکیبی) انجام پذیرفت. یافته‌ها: در بین 100 ایزوله جمع آوری شده، بیشترین میزان مقاومت به آمپی سیلین (85%) و هیچ مقاومتی به ایمی پنم دیده نشد. همچنین نتایج آزمون دیسک ترکیبی نشان داد که 80% ایزوله ها ESBLs مثبت می‌باشند. بحث و نتیجه‌گیری: نتایج این مطالعه نشان می‌دهدکه فراوانی ESBLs در شهرستان دلفان بالاست. لذا شناسایی ایزوله‌های ESBLs مثبت توسط آزمایشگاه‌های میکروب شناسی به روش دیسک ترکیبی، و تا حد ممکن، پرهیز از درمان تجربی بیماران بستری می تواند کارایی آنتی بیوتیک های بتالاکتام را ارتقاء بخشد.

Title: بررسی فراوانی علل تشنج در بیماران تشنجی مراجعه کننده به درمانگاه مغز و اعصاب شهرستان خرم آباد در سال 91-1390

http://yafte.lums.ac.ir/article-1-1665-fa.html

مقدمه: هدف اصلی مطالعه حاضر توصیف فراوانی علل تشنج در بیماران تشنجی مراجعه کننده به طور سرپایی به درمانگاه مغز و اعصاب شهرستان خرم آباد بود مواد و روش‌ها: این مطالعه در شهرستان خرم آباد انجام شد و پرونده بیماران مراجعه کننده در طی 15 ماه از اول فروردین 1390 تا آخر خرداد 1391 مورد بررسی قرار گرفت. از میان متغیرهایی که بررسی شده شیوع سنی و جنسی تشنج، وجود سابقه خانوادگی، وجود اختلال در سطح کلسیم خون، سدیم خون، قند خون، وجود سابقه عفونت مغزی، وجود سابقه تروما و تومور اهمیت ویژه ای داشت. یافته‌ها: از 221 بیمار مورد مطالعه 54% مرد و 46% زن بودند، شایع ترین گروه سنی درگیر، گروه 19-10 سال بودند که 34% موارد را شامل می شد، 7/7% بیماران سابقه خانوادگی مثبت داشتند، 45% بیماران اختلال در سطح کلسیم خون داشتند، 90% بیماران اختلال در سطح قند خون داشتند، هیچ بیماری اختلال در سطح سدیم خون نداشت، 90% موارد دارای سابقه عفونت مغزی داشتند، 7/7% بیماران سابقه ای از تروما داشتند، 6/75% بیماران صرع ایدیوپاتیک داشتند، 45% بیماران سابقه تومور داشتند و در 35/1% موارد علت تشنج در پرونده آنها ذکر نشده بود. بحث و نتیجه‌گیری: گرچه تشنج در بیشتر موارد اختلال خوش خیمی محسوب می شود، ولی فراوانی آن دسته از تشنج ها که نیازمند بررسی، تشخیص و برنامه ریزی درمانی ویژه هستند، آن قدر هست که در هر مورد شرح حال کامل، معاینه دقیق و ارزیابی پاراکلینیک انجام شود، زیرا ارزیابی تشخیصی روی تصمیمات درمانی، مشاوره خانوادگی، نیاز به پیگیری شدن و پیگیری خاص این بیماران تأثیر می گذارد.

Title: مقایسه شاخص توده بدنی قبل از بارداری با میزان اضافه ‌وزن بارداری بر دیابت بارداری در زنان باردار مراجعه‌کننده به بیمارستان عسلیان در سال‌ 92-1391

http://yafte.lums.ac.ir/article-1-1666-fa.html

مقدمه: علاوه بر ﺗﻐﺬﻳﻪ ﮐﺎﻓﻲ ﻭ ﺗﻌﺎﺩﻝ ﺑﻴﻦ ﻣﻮﺍﺩ ﻣﻐﺬﻱ ﺩﺭ ﺭﮊﻳﻢ ﻏﺬﺍﻳﻲ ﻣﺎﺩﺭ باردار ﺭﻭﻧﺪ ﺍﻓﺰﺍﻳﺶ ﻭﺯﻥ ﻣﺎﺩﺭ ﺩﺭ ﺩﻭﺭﺍﻥ ﺑﺎﺭﺩﺍﺭﻱ ﺑﺮ سلامت مادر و ﺍﻓﺰﺍﻳﺶ ﻭﺯﻥ ﺩﺭ ﺟﻨﻴﻦ ﺍﺛﺮ ﻋﻤﺪﻩﺍﻱ ﺩﺍﺭﺩ. هدف از این مطالعه مقایسه شاخص توده بدنی قبل از بارداری با میزان اضافه‌وزن دوره بارداری در بروز دیابت بارداری و ماکروزومی بر اساس تعریف جدید دیابت بارداری است. مواد و روش‌ها: نوع مطالعه مقطعی توصیفی ـ تحلیلی بود که بر روی زنان باردار 18 تا 35 ساله بدون ابتلا به بیماری زمینه‌ای انجام شد. نمونه‌گیری به‌صورت متوالی در دسترس انجام شد. در اولین ماه از بارداری وزن و قد زنان و فشارخون آن‌ها و همچنین قند خون ناشتای آن‌ها اندازه‌گیری شد. دومین ارزیابی در هفته‌های 28ـ24 بارداری به‌صورت اندازه‌گیری مجدد وزن و انجام تست غربالگری دیابت بارداری (GCT) انجام گرفت. سومین ارزیابی در پایان بارداری به‌صورت اندازه‌گیری وزن مادر و وزن نوزاد انجام شد. داده‌ها وارد نرم‌افزار SPSS شده و با استفاده از آزمون t مستقل و Mann-Whitney تجزیه و تحلیل شدند. یافته‌ها: در این مطالعه 600 زن باردار ارزیابی شدند. میانگین شاخص توده بدنی قبل از بارداری در زنان مبتلا به دیابت بارداری و مادران دارای نوزادان ماکروزوم، به‌طور معنی‌داری بیش از زنانی بود که عوارض فوق را نداشتند (0001/0=P). میانگین میزان اضافه‌وزن کسب‌شده در دوره بارداری در زنان مبتلا به دیابت بارداری و نوزادان ماکروزوم به‌ طور معنی‌داری بیش از زنانی بود که به عوارض فوق دچار نشدند (بترتیب 0001/0=P و 039/0=P). شاخص توده بدنی قبل بارداری در مقایسه با اضافه ‌وزن کسب‌ شده در دوره بارداری همبستگی بیشتری با دیابت بارداری داشت (278/0 در مقابل 077/0). بحث و نتیجه‌گیری: شاخص توده بدنی قبل بارداری در مقایسه با میزان اضافه ‌وزن دوره بارداری همبستگی بیشتری با دیابت بارداری و ماکروزومی دارد.

Title: سلامت روان زنان باردار مراجعه کننده به مراکز بهداشتی شهر خرم آباد

http://yafte.lums.ac.ir/article-1-1667-fa.html

مقدمه: در طی بارداری، تغییرات جسمی و روحی عمده‌ای در زنان باردار رخ می‌دهد و این دوره به عنوان یک دوره آسیب پذیر در زنان در نظر گرفته می شود. این مطالعه با هدف تعیین وضعیت سلامت روان زنان در دوران بارداری انجام شد مواد و روش‌ها: در این مطالعه توصیفی- مقطعی 280 زن باردار مراجعه کننده به 7 مراکز بهداشتی- درمانی شهر خرم آباد بررسی شدند. از روش نمونه گیری تصادفی ساده برای انتخاب مراکز بهداشتی- درمانی و روش نمونه گیری تصادفی نظام دار برای انتخاب زنان استفاده شد. ابزار مورد استفاده در این تحقیق پرسشنامه سلامت عمومی گلدبرگ بود. پس از جمع آوری داده ها با نرم افزار spss و آزمون تحلیل واریانس یک طرفه و آزمون t تجزیه و تحلیل گردید. یافته‌ها: نتایج نشان داد که 6/43% از زنان باردار از نظر سلامت روان مشکوک بودند. بالاترین میانگین نمرات مربوط به اختلال عملکرد اجتماعی(81/7) و کمترین مربوط به افسردگی(86/2) بود. در این مطالعه بین بارداری ناخواسته و جنسیت جنین با سلامت روانی زنان باردار ارتباط معناداری وجود داشت (05/0P<). همچنین میزان اضطراب زنانی که در سه ماهه سوم بارداری قرار داشتند نسبت به زنانی که در سه ماهه اول بارداری بودند به طور معناداری بالاتر بود (05/0P<). همچنین میزان اضطراب زنانی که در سه ماهه سوم بارداری قرار داشتند نسبت به زنانی که در سه ماهه اول بارداری بودند به طور معناداری بالاتر بود (05/0P<). بحث و نتیجه‌گیری: با توجه به نتایج این مطالعه و اهمیت سلامت روانی زنان باردار و تأثیرآن بر سلامت جنین و نوزاد، پیشنهاد می‌شود مشاوره روانپزشکی به عنوان جزیی از برنامه معمول مراقبت‌های بارداری مد نظر قرار گیرد.

Title: بیان پذیرنده‌های SDF1 در بیماران مبتلا به لنفوم هوچکین

http://yafte.lums.ac.ir/article-1-1668-fa.html

مقدمه: لنفوم هوچکین یک نوع بدخیمی سلول‌های سیستم ایمنی است و حدود 30% کل لنفوم‌ها را تشکیل می‌دهد. این بیماری براساس یافته‌های هیستوپاتولوژیک به چهار زیرگروه تقسیم می‌شود. گزارش های اخیر نشان می‌دهد که کموکاین SDF1 در رشد، بقا و متاستاز بسیاری از سرطان ها مثل سینه، پروستات و پانکراس نقش اساسی دارند اما تاکنون بیان آن و بویژه پذیرنده‌های آن یعنی CXCR4 و RDC1 در لنفوم هوچکین بررسی نشده است. لذا در این مطالعه برای نخستین بار بیان پذیرنده‌های SDF1 در بیماران مبتلا به لنفوم هوچکین مورد بررسی قرار گرفت. مواد و روش‌ها: در این مطالعه از بیوپسی‌های بدست آمده از 40 بیمار مبتلا به لنفوم هوچکین لام تهیه شد و با استفاده از روش ایمونوهیستوشیمی وجود پذیرنده‌های SDF1 مورد بررسی قرار گرفت. همچنین با مقایسه یافته‌های حاصل از ایمونوهیستوشیمی در بیان CXCR4 و RDC1 با اطلاعات بالینی بیماران ارتباط بین این مولکول‌ها با زیرگروه بیماری تعیین گردید. یافته‌ها: هر دو پذیرنده SDF1 در سطح سلول‌های هوچکین و Reed-Sternberg بیان می‌شوند. CXCR4 نه تنها در سطح سلول‌های هوچکین و Reed-Sternberg بلکه در سطح سلول‌های نرمال و واکنشی اطراف سلول‌های توموری نیز بیان می‌شود. ولی RDC1 تنها در سطح و داخل سیتوپلاسم سلول‌های توموری بیان می‌گردد. بحث و نتیجه‌گیری: نتایج این مطالعه نشان داد که RDCمی‌تواند به عنوان یک مارکر برای تشخیص لنفوم هوچکین مورد استفاده قرار گیرد. اگرچه مطالعات بیشتر با استفاده از حجم نمونه بزرگتر و تکنیک‌های دقیق‌تر جهت تأیید نتایج این مطالعه ضروری می‌باشند.

Title: مقایسه اثر بازدارندگی دو حالت اسانس و پودر خشک ریحان سبز بر جمعیت میکروبی گوشت چرخ شده گاو طی مدت نگهداری در یخچال

http://yafte.lums.ac.ir/article-1-1669-fa.html

مقدمه: ریحان با نام علمی basilicum Ocimum یکی از سبزیجات رایج معطر خوراکی است که در طب سنتی خواص مفیدی برای آن شناخته شده است. هدف از انجام این تحقیق در مرحله اول مقایسه قدرت ضد میکروبی اسانس روغنی ریحان سبز و ریحان بنفش در شرایط آزمایشگاهی جهت انتخاب نمونه واجد ترکیبات زیست فعال با پتانسیل کاربردی بیشتر و در مرحله بعد مقایسه اثر استفاده از دو حالت اسانس و پودر خشک نمونه انتخاب شده در گوشت چرخ شده گاو در ثبات جمعیت میکروبی و افزایش مدت ماندگاری آن در یخچال بود. مواد و روش‌ها: در بخش اول این تحقیق، قابلیت ضد میکروبی دو اسانس ریحان سبز و ریحان بنفش با روش انتشار دیسک بررسی شد. بر اساس نتیجه بدست آمده، در بخش دوم تحقیق اثر اسانس ریحان سبز در سطح 1/0 درصد و معادل پودر خشک آن ( 22درصد) در گوشت گاو خام به منظور بررسی جمعیت میکروبی کل در مدت 10 روز نگهداری در یخچال در مقابل نمونه شاهد ارزیابی شد. یافته‌ها: اسانس ریحان سبز در غلظت 20 میلی گرم برمیلی‌لیتر با تفاوت معنی‌داری (05/0P<) نسبت به اسانس ریحان بنفش در جلوگیری از رشد باکتری برتری داشت. این تفاوت در غلظت‌های 5 و 10 میلی‌گرم بر میلی‌لیتر نیز به شکل نسبی قابل مشاهده بود. در بخش دوم آزمایشات مشخص شد که استفاده از هر دو تیمار حاوی اسانس در سطح 1/0 درصد و پودر خشک گیاه ریحان سبز (22 درصد) در کاهش جمعیت میکروبی مؤثر بوده است. اگرچه پودر خشک در روز‌های آخر نگهداری خواص ضد میکروبی قوی‌تری را نسبت به اسانس نشان داد. بحث و نتیجه‌گیری: نتایج نشاد داد که هر دو حالت اسانس و پودر خشک گیاه ریحان سبز در افزایش پایداری میکروبی گوشت طی دوره نگهداری مؤثر بودند. با ذکر اینکه پودر خشک قابلیت نگهدارندگی بیشتری را در انتهای دوره نشان داد. بنابراین می‌توان از این مواد طبیعی به عنوان جایگزین نگهدارنده‌های سنتزی به منظور کاهش بیماری‌های مصرف کننده و بهبود عطر و طعم غذا استفاده کرد.

Title: مقایسه بین دو رژیم درمانی نگهدارنده و انفوزیون پیوسته سولفات منیزیوم وریدی در توقف دردهای زایمانی زودرس در خانم های باردار در سنین حاملگی 34-26 هفته

http://yafte.lums.ac.ir/article-1-1670-fa.html

مقدمه: هدف از انجام این مطالعه مقایسه اثربخشی دو رژیم درمانی نگهدارنده و انفوزیون پیوسته سولفات منیزیم در درمان زایمان زودرس در زنان حامله 34-26 هفته بود. مواد و روش‌ها: این کارآزمایی بالینی یک سو کور بر روی 70 زن باردار با تخصیص تصادفی انجام گرفت. در هر کدام از گروه ها با gr4 سولفات منیزیوم وریدی، در cc200 دکستروز 5% طی 20-15 دقیقه gr 20 سولفات منیزیوم وریدی طی 10 ساعت (با سرعت gr/hr2) بصورت انفوزیون وریدی (گروه A) و یا به مدت 12 ساعت سولفات منیزیوم gr/hr 2 به عنوان درمان نگهدارنده (گروه B) تجویز شد. اطلاعات بیماران جمع آوری و توسط نرم افزار SPSS 13 و با تست های آماری T test و Chi-Square مورد تجزیه و تحلیل واقع شد. P کمتر از 05/0 معنی دار در نظر گرفته شد. یافته‌ها: توقف دردهای زایمانی بعد از 24 و 48 ساعت، یک هفته، هفته 34 در گروه B در مقایسه با گروه A به طور معنی داری بیشتر بود. اما بین توقف دردهای زایمانی تا هفته 37 در دو گروه تفاوت معنی‌داری مشاهده نشد. تعداد روزهای حاملگی و آپگار دقیقه 5 نوزاد در گروه B بیشتر از گروه A بود و میزان بستری نوزادان در NICU در گروه B کمتر از گروهA بود. بحث و نتیجه‌گیری: رژیم درمانی نگهدارنده با سولفات منیزیوم از رژیم درمانی تزریق پیوسته آن در متوقف کردن دردهای زایمانی مؤثرتر است که برای جلوگیری از زایمان زودرس توصیه می شود.

Title: بررسی رابطه تقید به نماز و سلامت روان در دانشجویان دانشگاه پیام نور خرم‌آباد

http://yafte.lums.ac.ir/article-1-1671-fa.html

مقدمه: هدف اصلی این تحقیق بررسی رابطه تقید به نماز و سلامت روان در بین دانشجویان دانشگاه پیام نور خرم‌آباد می باشد. مواد و روش‌ها: روش تحقیق تحلیلی از نوع همبستگی است. جامعه تحقیق حاضر شامل کلیه دانشجویان دانشگاه پیام نور خرم‌آباد و نمونه‌ای به حجم 400 نفر براساس جدول مورگان با روش نمونه‌گیری طبقه‌ای متناسب از جامعه فوق انتخاب شد. برای گردآوری داده‌ها درباره تقید به نماز از پرسشنامه تقید به نماز پناهی (1382) و برای سنجش سلامت روان از فرم 28 سوالی پرسشنامه سلامت عمومی گلدبرگ و هیلیر (1979) استفاده شد. در مطالعه مقدماتی ضریب پایایی برای پرسشنامه تقید به نماز 906/0 و برای پرسشنامه سلامت عمومی ضریب پایایی 95/0 بدست آمد. در این تحقیق از نشانگرهای توصیفی (فراوانی، درصد و درصد تراکمی) و از آزمون استنباطی همبستگی گشتاوری پیرسون استفاده شد. یافته‌ها: نتایج تحقیق نشان دادند که 7/68 درصد از دانشجویان مورد مطالعه به طور خیلی زیاد، 5/26 درصد به طور زیاد، 8/3 درصد به طور متوسط، و فقط 1 درصد به طور کم و بسیارکم دارای تقید به نماز هستند. براساس یافته‌ها 5/47 درصد از نمونه مورد بررسی دارای هیچ یا کمترین حد اختلال در سلامت روان، 3/33 درصد از نمونه دارای اختلال در سلامت روان به طور خفیف، 5/14 درصد از نمونه دارای اختلال در سلامت روان به طور متوسط، 7/4 درصد از نمونه دارای اختلال در سلامت روان به طور شدید بوده‌اند. نتایج همچنین نشان دادند که همبستگی معنی‌داری بین تقید به نماز و سلامت روان هم در مقیاس کلی سلامت روان و هم در هر کدام از خرده مقیاسهای سلامت روان وجود دارد. بحث و نتیجه‌گیری: بنابراین با توجه به نتایج این پژوهش با اتخاذ روش های مناسب می توان به بالا بردن سطح سلامت روانی دانشجویان کمک نمود. اتخاذ برنامه های فرهنگی جهت تقویت تقیدی به نماز از پایه ابتدایی امکان بالا بردن سلامت روانی دانش آموزان را در سالهای بعد افزایش خواهد داد.

Title: بررسی میزان تأثیر آموزش براساس الگوی توانمندسازی فردی بر کیفیت زندگی زنان یائسه زرندیه

http://yafte.lums.ac.ir/article-1-1672-fa.html

مقدمه: اگر چه یائسگی قسمتی از فرایند طبیعی زندگی زنان است اما عوارض آن می تواند بر سلامت و کیفیت زندگی زنان مؤثر باشد، پژوهش حاضر با هدف تعیین تأثیر آموزش براساس الگوی توانمندسازی فردی بر کیفیت زندگی زنان یائسه زرندیه انجام شده است. مواد و روش‌ها: در این مطالعه نیمه تجربی، 90 نفر از زنان به روش تصادفی انتخاب و به دو گروه آزمون و کنترل (هر گروه 45 نفر) تخصیص یافتند. ابزار گردآوری اطلاعات، پرسشنامه کیفیت زندگی در دوران یائسگی بود. بر اساس اطلاعات به دست آمده از پیش آزمون برنامه آموزشی بر اساس الگوی توانمند سازی طراحی و اجرا شد. سه ماه بعد از مداخله، اطلاعات با استفاده از همان پرسشنامه اولیه جمع آوری و با استفاده از نرم افزار16 SPSS تجزیه و تحلیل شد یافته‌ها: قبل از مداخله آموزشی میانگین نمره کیفیت زندگی گروه آزمون 40/76 و گروه کنترل 38/72 بود که سه ماه بعد از مداخله به ترتیب به 48/68 و 76/71 تغییر یافت. همچنین بعد از مداخله، میانگین نمره سازه های الگوی توانمند سازی (عزت نفس و خودکارآمدی) در گروه آزمون در مقایسه با گروه کنترل افزایش یافت. آزمون آماری نشان دهنده تغییر معنی دار نمره کیفیت زندگی و سازه های الگوی توانمند سازی در گروه آزمون بود (001/0P<) . بحث و نتیجه‌گیری: یافته ها از تأثیر مثبت برنامه آموزشی طراحی شده بر اساس الگوی توانمند سازی بر کیفیت زندگی پشتیبانی می کند. لذا طراحی و اجرای برنامه های آموزشی مشابه به منظور بهبود کیفیت زندگی زنان یائسه در مناطق دیگر توصیه می‌شود.

Title: اثرات عصاره هیدروالکلی گیاه تیمبرا بر باکتری‌های گرم مثبت و منفی بیماری‌زا

http://yafte.lums.ac.ir/article-1-1673-fa.html

مقدمه: درمان بیماری‌های باکتریایی توسط آنتی بیوتیک‌های سنتتیک با بروز مشکلاتی از قبیل ایجاد عوارض جانبی در انسان‌ها و ایجاد مقاومت در میکروارگانیسم‌ها همراه است. اخیراً به استعمال گیاهان در درمان بیماری‌های باکتریایی توجه زیادی شده است. از آنجایی که استفاد از گیاه تیمبرا اسپیکاتا در طب سنتی ایران مرسوم است، در این تحقیق اثر ضد باکتریایی عصاره این گیاه بر روی تعدادی از باکتری های بیماری‌زا بررسی شد. مواد و روش‌ها: گیاه مذکور از کوههای زاگرس شهر ایلام جمع آوری و پس از شناسایی و نامگذاری، عصاره به روش ماسراسیون تهیه شد. غلظت‌های مختلف عصاره با استفاده از روش دیسک دیفیوژن بر روی باکتری‌ها تأثیر داده شد. از آنتی‌بیوتیک‌های تشخیصی به عنوان کنترل مثبت و از DMSO به عنوان کنترل منفی استفاده گردید. میزانMIC و MBC نیز مشخص شد. یافته‌ها: بیشترین تأثیر عصاره در باکتری های گرم مثبت دیده شد. بیشترین قطر هاله در غلظت mg/ml 76 مشاهده، و در این میان S. aureus و S. epidermidis بیش از سایر میکروارگانیسم‌ها حساس بودند (05/0P<). کمترین میزان MIC مربوط بهS. aureus 1885 با میزان mg/ml5/2 و S. epidermidis 2405 با mg/ml5 بوده و E. coliو K. pneumonia با 15 میلی‌متر قطر هاله عدم رشد، حساس-ترین باکتری‌های گرم منفی به عصاره بودند (05/0P<). بحث و نتیجه‌گیری: عصاره هیدروالکلی تیمبرا تأثیر ضد باکتریایی قابل ملاحظه‌ای داشت. بررسی قطر هاله‌های عدم رشد نشان داد که عصاره در غلظت‌های مورد استفاده بر باکتری‌های گرم مثبت مؤثرتر بوده است.

Title: مقایسه میزان تولید پروتئین نوترکیب در دو رده سلولی CHO و MCF7

http://yafte.lums.ac.ir/article-1-1674-fa.html

مقدمه: هدف ازمطالعه‌ی حاضر، مشخص نمودن رده سلولی مناسب‌تر، جهت مطالعات تولید پروتئین در سیستم‌های یوکاریوتی می‌باشد. پس از ترانسفکشن، تغییرات ایجاد شده در سطوح بیانیRNA و پروتئین نوترکیب هدف ارزیابی شد. مواد و روش‌ها: به منظور بررسی بیان ژن مورد نظر و تولید پروتئین نوترکیب E6 پاپیلوما ویروس انسانی تیپ 16 در سلول‌‌های مورد آزمایش، از پلاسمید نوترکیب pcDNA3-E6 و نیز دو رده سلولی متفاوت MCF7 و CHO استفاده گردید. به این منظور به دنبال ترانفکشن، بیانRNA و پروتئین هدف بوسیله واکنش زنجیره‌ای پلیمراز معکوس (RT-PCR) و آزمایش لکه‌گذاری وسترن با استفاده از ژل SDS-PAGE و مونوکلونال آنتی بادی Anti-E6 ارزیابی گردید. یافته‌ها: در این مطالعه به دلیل غلظت بسیار پایین پروتئین تولیدی در سلول‌های MCF7 ، آزمایش RT-PCR مثبت و آزمایش وسترن بلاتینگ آن منفی شد، این در حالی است که در رده سلولی CHO هر دو آزمایش ذکر شده مثبت شدند بحث و نتیجه‌گیری: با توجه به نتایج بدست آمده توصیه می‌شود از سلول CHO به منظور ترانسفکشن و تولید پروتئین نوترکیب در سیستم‌هایی که تولید بالای پروتئین امکان پذیر نیست استفاده گردد
